# A System Genito-Urinary Diseases, Syphilology and Dermatology

**Published:** 1894-01

**Authors:** 


### Book Reviews.

A SYSTEM OF GENITO-URINARY DISEASES, SYPHILOLOGY AND DERMATOLOGY, —By various Authors.—Edited by Prince A. Morrow, A. M., M. D. Published by D. Appleton & Co., New York. Sold by Subscription only.

The second volume of Morrow's system of Genito-Urinary Diseases, Syphilology and Dermatology, is devoted to a consideration of syphilis and chancroid.

Most of the contributors are well-known authors and have presented their veiws and conclusions, largely the result of long and valuable experience, with a clearness and fullness that leaves little to be desired. While the work is eminently adapted to the needs of the specialist in this province, it was nevertheless no class restriction, as is evinced by the brilliant chapters on syphilis of the viscera, of the nervous system, of the air-passages, etc., so that it thus meets the requirements of the general practitioner.

The relation of syphilis to the public health, to marriage, its demographic relations, and means of repression, have been discussed at length by Dr. Samuel T. Armstrong.

The therapy of syphilis is very complete and finds place both in relation to local and individual lesions and in a geneaal summary, near the end of the volume, in which such therapeutic procedures as are of value are considered, while those that are obsolete and the outcome of painful theories receive no mention.

The volume is not encumbered by that profusion of bibliographical reference which is so frequent and which serves no purpose other than to draw an unwarranted attention to the author's erudition. The references have been embodied largely in the text—their proper place when they elucidate rather than offend.

The text is embellished by numerous well-executed illustrations, typogravures, half-tone and cromo lithographic plates, together with others which have been gotten by a combination of photography and lithography. Tnese latter are claimed by the publishers to be unique features of the volume.

I am glad to note the absence of those grotesque charactera-tures of reality—those old wood-cuts, which seem to be common property and which have so long disfigured the pages of works on surgery and venereal diseases. B. W.

